# Intraperitoneal lipoma: A case report

**DOI:** 10.1016/j.ijscr.2025.111443

**Published:** 2025-05-14

**Authors:** Ben Marzouk Saoussen, Rabti Souphia, Farjaoui Wael, Ben Hassine Basma, Mighri Roua, Khalifa Mohamed Bechir

**Affiliations:** a General Surgery Department, Military Hospital of Tunis, Mont Fleury-1008, Tunis, Tunisia; b Faculty of Medicine of Tunis, 15, Djebel Lakhdhar Street – 1007 Bab Saadoun, Tunis, Tunisia; c Faculty de Medecine Ibn El Jazzar of Sousse, Mohamed Karoui Street - 4002, Sousse, Tunisia

**Keywords:** Omental lipoma, Intraperitoneal lipoma, Benign tumor, Abdominal mass, Surgical excision, CT imaging, MRI imaging, Histopathology

## Abstract

**Introduction:**

Lipoma is a very common benign tumor of diverse localization, formed by mature adipocyte cells without cyto-nuclear atypia. Intra-abdominal localization, particularly from omental origin, is uncommon and can present with diffuse abdominal pain, intestinal occlusion, or perforation.

**Case report:**

We present the case of a 43-year-old man (BMI 24.8, normoglycemic, normal lipid profile) with right hypochondrial pain and a palpable abdominal mass. Contrast-enhanced CT and MRI revealed a fatty intraperitoneal mass measuring 14 cm in length, originating from the greater omentum. Fine needle aspiration cytology (FNAC) was not performed preoperatively due to the highly suggestive benign imaging features. Surgical exploration confirmed an 8 cm lipomatous mass adherent to the greater omentum, which was completely resected. Histopathological examination confirmed a lipoma without malignancy.

**Discussion:**

Omental lipomas represent a subset of intraperitoneal adipose tumors, primarily affecting adults aged 40–60. While both contrast-enhanced CT and MRI aid in diagnosis, MRI provides superior tissue characterization for fat-containing lesions. Histology remains essential to rule out liposarcoma, with core needle biopsy indicated for lesions with concerning features. Surgical removal with capsule preservation minimizes recurrence risk, which remains low (2–5 %) but warrants long-term surveillance.

**Conclusion:**

Omental lipomas are uncommon, usually asymptomatic, and require imaging for diagnosis and surgery for definitive treatment. Surveillance imaging is recommended to monitor for recurrence.

## Highlights

• Intraperitoneal lipomas are rare and typically asymptomatic.
• Imaging aids diagnosis, but histological confirmation is vital to exclude malignancy.
• Complete surgical excision is the treatment of choice, with low recurrence rates.
• Regular postoperative surveillance is essential for early detection of recurrence or potential malignant transformation.

## 1 Introduction

Lipomas are benign tumors composed of mature adipose cells [1]. They are frequent mesenchymal tumors of undetermined etiopathogenesis. Omental and mesenteric lipomas remain relatively uncommon localizations, with fewer than 50 cases of omental lipomas reported in the literature according to our review [2,3]. They are most often asymptomatic or discovered when a complication such as small bowel volvulus occurs. Symptoms vary according to the size of the lipoma and its location in the peritoneal cavity [2,3]. Contrast-enhanced CT scan and MRI are valuable in establishing the diagnosis and ruling out malignancy. The treatment of choice is surgical excision.

We report the case of an omental lipoma discovered in a patient with right hypochondrium pain.

This work has been reported in line with the SCARE 2023 criteria [4].

## 2 Case report

A 43-year-old male patient (height: 175 cm, weight: 76 kg, BMI: 24.8) with no significant medical history presented with abdominal pain in the right hypochondrium and right flank. He had no history of diabetes, obesity, or hypercholesterolemia, confirmed by normal fasting blood glucose (5.1 mmol/L) and lipid profile (total cholesterol: 4.8 mmol/L, LDL: 2.9 mmol/L, HDL: 1.2 mmol/L, triglycerides: 1.5 mmol/L).

Clinical examination revealed a palpable mass in the right flank, soft, well-limited, and painless, measuring approximately 6 cm in diameter. Ultrasound suggested an intraperitoneal fatty mass. Contrast-enhanced CT scan confirmed a 14 cm long homogeneous intraperitoneal fatty mass with a density between −80 and −120 Hounsfield units, without septations, calcifications, or enhancement after contrast administration, suggestive of a benign lipoma.

**Fig. 1:**
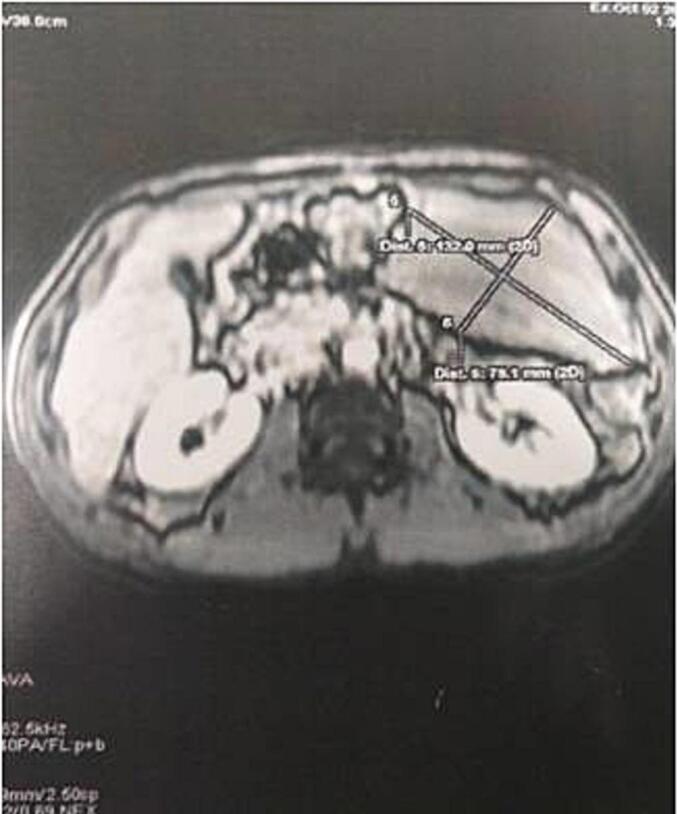
Abdominal MR showing intra-peritoneal mass on the left flank, with a homogeneous fatty signal, suggesting in the first instance an intra-peritoneal lipoma.

An abdominal MRI was ordered for better tissue characterization, which demonstrated an intra-peritoneal mass in the left flank with homogeneous high signal intensity on T1-weighted images, signal dropout on fat-suppression sequences, and no enhancement after gadolinium administration, consistent with an intra-peritoneal lipoma originating from the greater omentum (Fig. 1).

Preoperative tissue biopsy was not performed due to the characteristic imaging features strongly suggesting a benign lipoma without any concerning features for malignancy. Additionally, potential track seeding in the rare case of a malignant lesion was considered in the decision-making process.

**Fig. 2:**
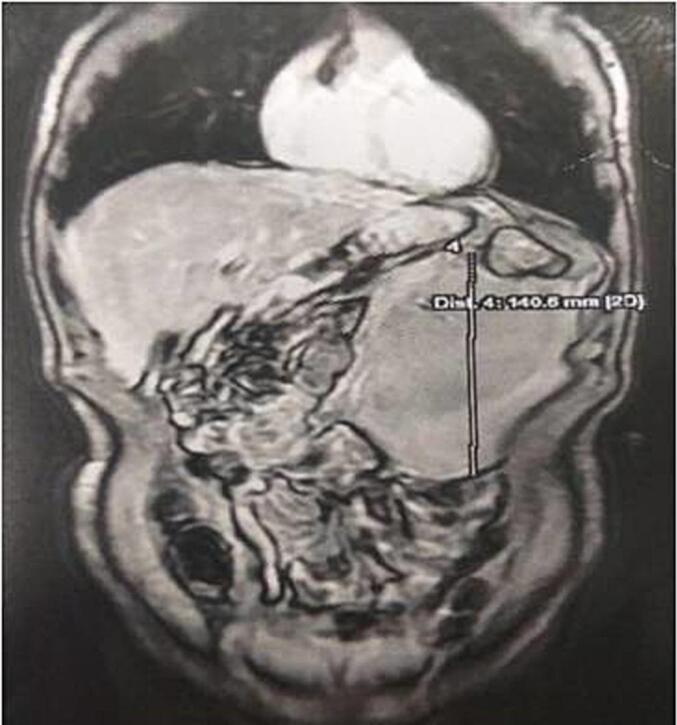
Per operative findings, a fatty mass measuring 8 cm in its long axis was identified, partially adherent to the greater omentum.

The patient underwent surgery via a median supra-umbilical approach. Surgical exploration revealed a fatty mass with a long axis of 8 cm, partially adherent to and clearly originating from the greater omentum. No adhesions or compression of neighboring organs were observed (Figs. 2 + 3).

**Fig. 3:**
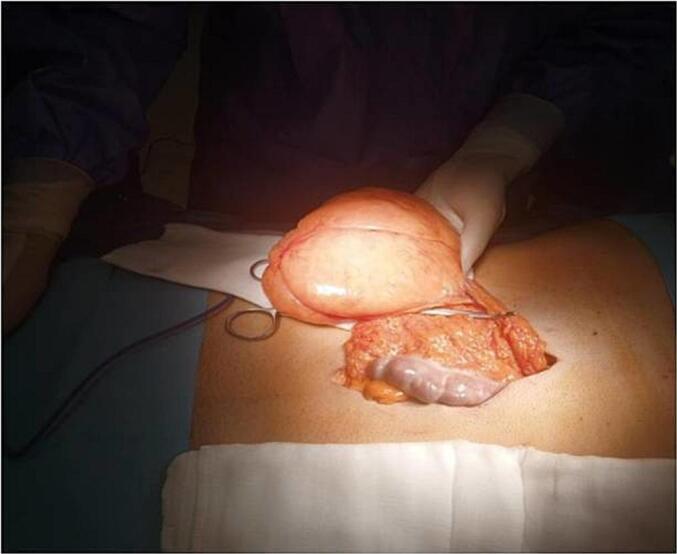
Fatty mass after resection.

**Fig. 4:**
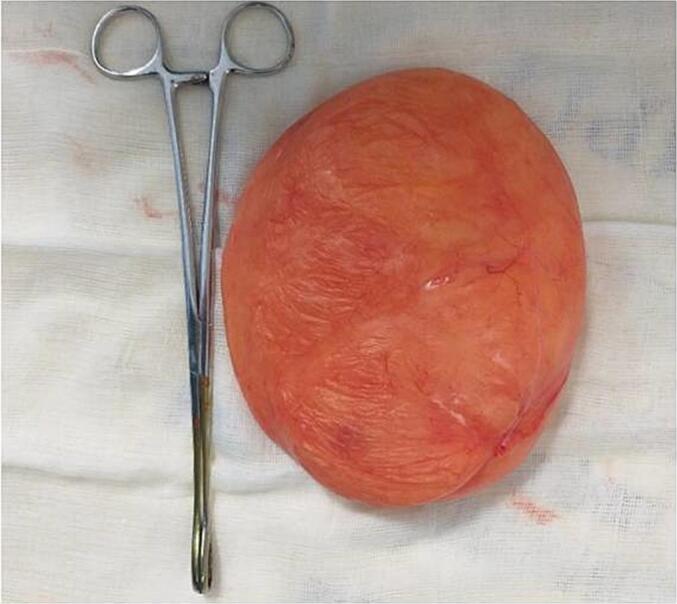
Histological appearance of the surgical specimen showing a lipoma composed of mature adipocytes without atypia or mitotic activity.

The mass was completely resected with preservation of the capsule. Histopathological examination confirmed a lipoma composed of mature adipocytes without atypia or mitotic figures, and no signs of malignant degeneration (Fig. 4).

## 3 Discussion

Lipomas are benign tumors affecting adults between the ages of 40 and 60, with no gender predominance [5]. Omental lipomas represent a small subset of intraperitoneal lipomas, with our literature review identifying fewer than 50 documented cases.

They are often clinically asymptomatic. Symptoms include palpable abdominal masses, abdominal pain, intestinal occlusion, volvulus, or intestinal perforation [6]. While lipomas are generally more frequent in diabetic, obese, and hypercholesterolemic subjects [6], our patient had none of these risk factors.

Radiological investigations play a crucial role in diagnosis. Contrast-enhanced CT scan can effectively characterize these lesions by demonstrating a homogeneous fatty mass (density between −80 and −120 Hounsfield units) without septations, calcifications, or enhancement after contrast administration [7]. MRI provides superior tissue characterization compared to CT, particularly for fat-containing lesions, with typical findings including high signal intensity on T1-weighted images, intermediate signal on T2-weighted images, and signal dropout on fat-suppression sequences [8]. However, definitive differentiation from liposarcoma requires histological examination [7].

In cases where imaging reveals concerning features such as thick septa (>2 mm), nodular or globular non-adipose areas, or enhancement after contrast administration, preoperative biopsy should be considered [9]. Core needle biopsy is preferred over fine-needle aspiration cytology (FNAC) for adipose tissue lesions as it provides better architectural information. However, negative biopsies do not definitively exclude malignancy due to potential sampling error [10].

Surgical treatment involves complete removal of the lipoma without rupture of the capsule, by laparotomy or laparoscopy depending on the size and location of the mass. Recurrence is possible in 2–5 % of cases, so prolonged surveillance with annual imaging (CT or MRI) for at least 5 years is recommended [11]. Repeated resections are thought to favor transformation of the lipoma into a liposarcoma [12].

## 4 Conclusion

Omental lipomas represent an uncommon subset of intraperitoneal lipomas. They typically remain asymptomatic until they reach a significant size or cause complications. Contrast-enhanced CT and MRI are invaluable diagnostic tools, with MRI offering superior tissue characterization. Surgical excision remains the treatment of choice, with long-term surveillance recommended due to the small risk of recurrence.

## Author contribution

Rabti Souphia and Ben Marzouk Sawssen contributed to manuscript writing and editing, and data collection; Wael Farjaoui, Ben Hassine Basma and Roua Mighri contributed to data analysis; Med Bachir Khalifa contributed to conceptualization and supervision; All authors have read and approved the final manuscript.

## Consent

Written informed consent was obtained from the patient for the publication of this case report and its accompanying images. A copy of the written consent is available for the Editor-in-Chief of this journal to review upon request.

## Ethical approval

Ethical approval is not applicable/waived at our institution. Due to the specific nature of case reports, which involve detailed descriptions of observations and interventions that have already been conducted on patients, as opposed to prospective studies involving planned interventions, our institution does not require formal ethical approval for such cases. We recognize the importance of ethics in medical research and are fully committed to upholding ethical standards in our medical and research practices.

## Guarantor

Rabti Souphia.

## Research registration number

N/A.

## Funding

This research did not receive funding from any specific grant provided by public, commercial, or not-for-profit organizations.

## Conflict of interest statement

No conflicts of interest.
